# The effect of orthodontic treatment on the periodontium and soft tissue esthetics in adult patients

**DOI:** 10.1002/cre2.480

**Published:** 2021-09-07

**Authors:** Reem S. Abdelhafez, Ahmad A. Talib, Dafi S. Al‐Taani

**Affiliations:** ^1^ Department of Preventive Dentistry Jordan University of Science and Technology Irbid Jordan

**Keywords:** esthetics, orthodontics, periodontium, soft tissue

## Abstract

**Objective:**

Most patients seek orthodontic treatment to achieve an esthetic outcome. Orthodontic treatment has possible negative sequelae. The aim of this study is to assess these possible effects on the periodontium and tissue esthetics.

**Methods:**

One hundred fifty‐six patients who have completed orthodontic treatment at Jordan University of Science and Technology clinics were recruited. They were divided into extraction and nonextraction subgroups. Another 155 patients never undergoing orthodontic treatment were assessed. The height of papilla, width of keratinized gingiva, gingival recession, degree of tooth display, smile line, crestal bone level, and proximal caries were assessed. Chi‐ square test was used for categorical/discrete variables while independent t‐test was used for continuous variables. The level of significance was set at (*p* ≤ 0.05).

**Results:**

The mean age was 22 years with no significant difference between the groups. There was a significant difference between “ortho” and “nonortho” groups in tooth display and keratinized gingiva (*p* = 0.006 and <0.001, respectively). The overall crestal bone level, smile line, recession, and papilla fill did not show any significant differences (*p* = 0.200, 0.067, 0.120, and 0.066, respectively). The crestal bone level in the upper and lower anterior segments was significantly lower in the “ortho” treated group compared to the “nonortho” treated group (*p* = 0.002 and 0.005, respectively). A significant difference between “extraction” and “nonextraction” groups was in the width of keratinized gingiva (*p* = 0.003) and the number of teeth displayed (*p* < 0.001). Despite reaching statistical significance these differences are not necessarily of clinical significance.

**Conclusion:**

Orthodontic treatment clearly affects the periodontal tissues; however, the detrimental effects appear to be minimal. Patients with history of orthodontic treatment might have lower crestal bone levels at certain sites and this should not be confused with periodontal disease.

## 1 INTRODUCTION

Nowadays adults have more desire to seek orthodontic treatment to reach the optimal esthetic appearance and function for their teeth. In fact, this makes decision‐making in orthodontic treatment a huge challenge for both periodontists and orthodontists and it raises the need for evidence‐based therapeutic concepts and a well‐designed treatment plan for each diagnosed periodontal status. According to Cardaropoli and Gaveglio (2007), orthodontic therapy is based on the principle of displacing teeth by applying mechanical forces and remodeling the periodontal ligament and alveolar bone. Periodontal ligament could go through reorganizing and remodeling due to orthodontic forces during tooth movement. A favorable tissue response will be produced by optimal orthodontic forces, but once the forces exceeded its magnitude or if the initial periodontal support is reduced then tissues will respond differently to movement forces (Cardaropoli & Gaveglio, 2007).

According to Fontenelle (1982), clinical, cellular, and molecular changes in the alveolar bone are detected after orthodontic treatment. In addition, the alveolar bone density was found to decrease. This was reported by Huang et al. (2013) and Hsu et al. (2011) who assessed the density of maxillary anterior segment. On the other hand, Patil et al. (2012) reported increased density of the alveolar bone. This might be due to changes in patient's age, gender, race (Chen et al., 2005), and other systemic variables (White & Rudolph, 1999).

An esthetically unpleasing appearance could be due to gingival recession which could be associated with the possible negative sequalae of root exposure such as root caries and sensitivity (Kassab & Cohen, 2003). It is commonly noticed that orthodontic movement of teeth outside the alveolar plate is a possible etiological factor for gingival recession (Wennström et al., 1987). Yared et al. (2006) reported that the height of the keratinized gingiva might be reduced post orthodontic treatment which will result in gingival recession. The integrity of the dento‐gingival junction must be maintained by the maximum amount of attached gingiva that will have a positive impact of reducing the incidence of developing gingival recession around teeth (Farnoush & Schonfeld, 1983).

Facial esthetics is a reason that mostly motivates patients to seek orthodontic treatment and other dental procedures (Andrews, 2008). Evaluation of facial esthetics, should include both tooth alignment and occlusion and an evaluation of the soft tissue–hard tissue relationship (Holdaway, 1983).

Tooth display is another factor added to this evaluation; an esthetic smile generally is the one which displays more teeth (Johnson & Smith, 1995).

Interdental papilla has two different possible scenarios when measured post orthodontic treatment. As gingival recession is one of the possible outcomes of orthodontic movement especially in the anterior teeth, this will play a negative role on reducing interdental papilla in those teeth (Andrews, 2008; Cardaropoli et al., 2001; Corrente et al., 2003; Duncan, 1997; Melsen, 1986; Melsen et al., 1989; Murakami et al., 1989; Rabie et al., 1998; Re et al., 2002). On the other hand, adequate orthodontic therapy might stimulate interdental papilla formation as realignment of adjacent teeth will form an adequate contact point in a manner which initiate an anatomical basis that serve interdental papilla formation (Cardaropoli & Re, 2005; Dorsey & Korabik, 1977; Kilpeläinen et al., 1993).

One third of all malocclusions require extraction of teeth to achieve the maximum acceptable outcome following orthodontic treatment as reported in the literature (Proffit & Fields, 2000). Debate has always been there to discuss the effect of these extractions, especially on factors that might affect facial esthetics of the patient.

In this study, several periodontal parameters that might be affected by orthodontic treatment will be assessed. These include crestal alveolar bone level, papilla height, width of the keratinized gingiva, the prevalence of gingival recession, proximal caries, and smile esthetics.

## 2 MATERIALS AND METHODS

The study was conducted in the Postgraduate Dental Clinics (PDC) and was approved by the Institutional Review Board. The participants were selected from patients attending the Dental Teaching Clinics at Jordan University of Science and Technology, and were either subjects who had completed orthodontic treatment at least 6 months ago or control subjects who are not indicated for orthodontic treatment having no malocclusion. All patients received detailed description of the planned treatment and informed consents were signed.

The sample size was calculated. This was determined using the minimum number per group of patients required using the Steven Thompson formula for sample size in a comparative study. The prevalence data from previously published studies were used, where the population (N) was considered to be 1700 subjects, the confidence level set at 95%, error proportion at 0.05, and probability at 50%. The primary outcome variable is periodontal health expressed by multiple clinical and radiographic parameters. The sample size was determined by estimating that the population of patients undergoing orthodontic treatment at the Dental Teaching Clinics at Jordan University of science and Technology during the period of the study to be 1700 patients. As recommended for cross‐sectional studies a sample size of 10% of the population and not exceeding 1000, the sample size of the “ortho” subjects was calculated to be approximately 170. A comparable sample of 170 subjects who have never undergone orthodontic treatment (“nonortho” group) was set resulting in a total sample of 340 subjects. Upon recruitment of the subjects taking into account the inclusion and exclusion criteria a study sample of 311 subjects (156 “ortho” subjects and 155 “nonortho” subjects were eligible and included in the study). The study population consisted of 311 adult patients (249 female and 62 male) with an age ranging between 18 and 39 years, attending the dental teaching center for a variety of reasons. The test group consisted of 156 adult patients who have completed orthodontic treatment. This test group was divided into an extraction subgroup; those who had extraction of the first or second premolars done as part of their orthodontic treatment, and a nonextraction subgroup. A matching control group of 155 patients who have never undergone orthodontic treatment were recruited from the same district. Both groups met the inclusion and exclusion criteria. Comparative qualitative methods were used to compare patients who had history of orthodontic treatment, and control subjects who never underwent any orthodontic tooth movement and were not indicated for orthodontic treatment.

Subjects included were 18 years or older with no systemic diseases or conditions. Periodontal assessment had to show absence of sites with probing depths >3 mm and of clinically detectable signs of inflammation. Subjects included in the control group had normal occlusion or slight malocclusion that was not to be treated orthodontically. The test group had normal occlusion or slight malocclusions after the conclusion of orthodontic treatment of different types of malocclusion.

Smokers, subjects noncompliant with oral hygiene measures, subjects exhibiting interdental clinical attachment loss [that is detectable at two or more nonadjacent teeth and buccal or oral clinical attachment loss of >3 mm with probing depth of >3 mm that is detectable at two or more teeth (Chapple et al., 2018)], subjects with history of treated periodontitis (health on a reduced periodontium) were excluded from the study and subjects exhibiting moderate to severe malocclusion were excluded. In addition, exclusion criteria included subjects with systemic conditions affecting the periodontal tissues, pregnant, and lactating mothers, Subjects with orthodontic appliances and subjects with extracted anterior or premolar teeth for nonorthodontic reasons.

All subjects were interviewed and screened by a single, experienced dentist with respect to their demographic data, age, sex, occupation and education status. Full medical and dental history and full mouth examination were carried out by the same dental practitioner, followed by two bitewing and two upper and lower anterior periapical radiographs taken by a radiologist.

Clinical Parameters assessed included the papilla fill; measured as the distance between the tip of the papilla and the contact point (Tarnow et al., 1992) and recession as the distance from the cemento‐enamel junction to the edge of the free gingival margin (Glavind et al., 1968) at the mid‐labial aspect. Both parameters were assessed for all teeth except third molars. The width of keratinized gingiva (KG) from the gingival margin to the mucogingival junction was measured at the mid‐labial aspect of upper and lower incisors and molars. In addition, assessment of tooth display when the mouth is relaxed and slightly open (Bhuvaneswaran, 2010) was from the inferior border of the upper lip to the incisal edge of the upper teeth and Smile line from the zenith of the gingival margin to the lower border of the upper lip (Van der Geld et al., 2011). The crestal bone level (CBL) was measured on radiographs at the mesial and distal surfaces of all teeth as the distance from the cemento‐enamel junction to the crest of the bone using a metal ruler (Kennedy et al., 1983), and proximal caries appearing as a radiolucency detected on the X‐rays between two adjacent teeth (Kamburoğlu et al., 2012). All radiographs were taken using the same X‐ray machine and by the same radiography technician using holders. Measurements were done by a single operator and using the same metal ruler. Magnification for the X‐ray machine was not assessed specifically for this research but when tested for other purposes was found to be 1:1. All clinical parameters were measured using Michigan O periodontal probe with Williams grading.

Data were entered and analyzed by the Statistical Package for Social Sciences (SPSS) software version 11.0 (SPSS®, Inc., Chicago, IL, USA). Shapiro–Wilk test was used to check the normality of data. The variables were normally distributed, therefore parametric tests were used. Frequencies, means, *SD*, and cross tabulations were chosen to facilitate description of the data. In addition, chi‐square test was used for categorical/discrete variables. The level of significance was set at (*p* ≤ 0.05).

## 3 RESULTS

### 3.1 Population of sample

A total of 311 subjects were recruited in this study, they were categorized in two main groups (ortho‐treated and nontreated). The age range of the whole population sample was between 18 and 39 years, according to the descriptive analysis the mean age of the subjects was around 22 years old. Females were more than 80% of the whole subjects (Table 1). No statistically significant variation was found among age and sex for “ortho‐treated” and “nontreated” groups. Apparently, one half of the study sample (50.1%) had ortho treatment. The percentage of “ortho‐treated” subjects who completed orthodontic treatment <12 months to the date of assessment was around (59.5%) of the “ortho‐treated” group. Moreover (81.1%) of that group did not extract any teeth for orthodontic purpose. (Table 2).

**Table 1:** Distribution of socio‐demographic variables of whole study sample (N = 311)

Variable^a^	Mean ± *SD* N(311)	Groups		*p*‐value
		Nontreated N(155)	Ortho‐treated N(156)	
Age (years)	21.47 ± 3.5	21.81 ± 3.8	21.14 ± 3.1	0.188**
Gender				0.713***
Female	249(80.1)	123(79.4)	126(80.7)	
Male	62(19.9)	32(20.6)	30(19.3)	

^a^ Socio‐demographic variables contains (patient's age, patient's gender).

^**^ *t*‐test,***χ ^2^‐test.

**Table 2:** Distribution of dental variables of whole study sample (N = 311)

Variable^a^	N(%)
Did they have orthodontic treatment	
No	155(49.8)
Yes	156(50.1)
When completed (months)	
≤12	62(40.5)
>12	94(59.5)
(mean ± *SD*)	33.97 ± 32.95
Range	168–6 = 162
Extraction	
No	252(81.1)
Yes	59(18.9)

^a^ Dental variables consists of (did study sample had orthodontic treatment, since when did the orthodontic treated group completed orthodontic treatment, extractions in the whole study sample).

### 3.2 Clinical parameters for “ortho” and “nonortho” groups

Table (4.3) shows the clinical parameters in both “ortho‐treated” and “nontreated” groups. A statistically significant difference was detected in some of the clinical parameters between those two different groups such as in tooth display length and the width of keratinized gingiva (*p* = 0.006, <0.001 respectively).

#### 3.2.1 Prevalence and degree of smile line and tooth display in the “ortho‐treated” and “nontreated” groups

The mean scores of smile line were around (1.05 mm) as shown in (Table 3) with higher smile line in “ortho‐treated” group being (1.15 ± 0.95 mm) with no significant difference (*p* = 0.067).

**Table 3:** Means (±*SD*) and N (%) of clinical parameters of whole study sample by groups (N = 311)

Variable^a^	Overall mean ± *SD*	Groups		*p*‐value independent *t*‐test
		Nontreated mean ± *SD*	Ortho‐treated mean ± *SD*	
No. of extracted teeth	0.49 ± 1.14	0.08 ± 0.37	0.9 ± 1.5	<0.001
Smile line	1.05 ± 0.92	0.96 ± 0.87	1.15 ± 0.95	0.067
Tooth display length	1.98 ± 0.97	1.83 ± 0.78	2.13 ± 1.11	0.006
Gingival recession	0.07 ± 0.11	0.06 ± 0.11	0.08 ± 0.11	0.120
Keratinized gingiva	2.89 ± 0.44	2.98 ± 0.47	2.79 ± 0.39	<0.001
No. displayed teeth	5.85 ± 1.61	5.93 ± 1.64	5.78 ± 1.57	0.410
**Papilla fill**	**N (%)**	**N (%)**	**N (%)**	*χ* ^2^–test 0.066
Reduced	150(47.9)	82(52.9)	68(43.0)	
Fully filled	163(52.1)	73(47.1)	90(57.0)

^a^ Mean of clinical parameters consist of (number of extracted teeth, smile line, length of tooth display, gingival recession, keratinized gingiva, number of displayed teeth and papilla fill) between nontreated and ortho‐treated groups.

**Figure 1:**
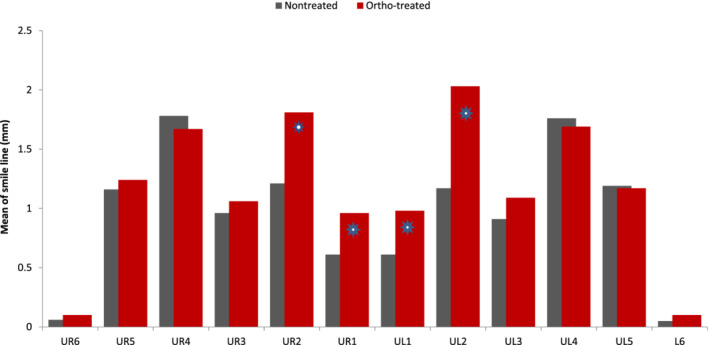
Mean of smile line for the sample population by tooth among treatment status

Furthermore, the highest smile line was at the lateral incisors then at the canines in the “ortho‐treated” group. On the other hand, higher smile line was shown in the first premolar in the “nontreated” group (Figure 1).

Smile line exhibited a statistically significant difference in both upper right and left central incisors showing a mean of (0.96 and 0.98 mm, respectively) for “ortho‐treated” group, this is in relation to both centrals of the “nontreated” group having a mean of (0.61 mm) for both upper right and left central incisors (Figure 1).

Statistically significant difference was obvious with regards to the length of tooth display between the two groups (*p* = 0.006), as shown in (Table 3). The overall mean of tooth display length was (1.98 ± 0.97 mm), showing (2.13 ± 1.11 mm) in “ortho‐treated” group compared to much lower mean of (1.83 ± 0.78 mm) in “nontreated” group.

The overall number of displayed teeth had a mean of (5.85 ± 1.61). “Nontreated” group showed higher number of displayed teeth with a mean of (5.93 ± 1.64) compared to fewer teeth displayed in “ortho‐treated” groups having a mean of (5.78 ± 1.57). Such variables between the different groups were not a statistically significant (*p* = 0.410) (Table 3).

#### 3.2.2 Prevalence and degree of gingival recession in the “ortho‐treated” and “nontreated” groups

**Figure 2:**
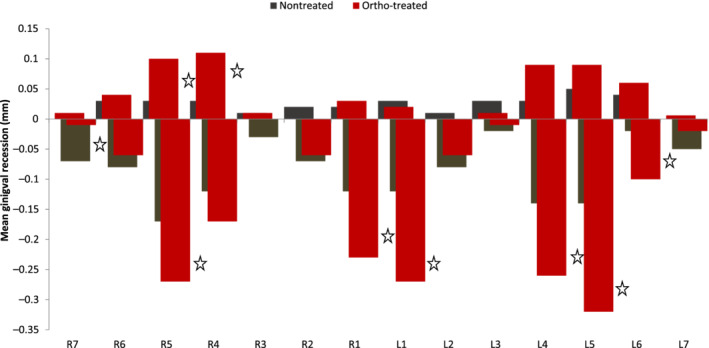
Mean of gingival recession for the sample population by tooth among treatment status

The mean scores of gingival recessions were around (0.07 mm) as shown in (Table 3) with more gingival recession in “ortho‐treated” group which did not reach statistical significance as shown in (Figure 2) (*p* = 0.120). Moreover, most of the gingival recession was evident at the lower second premolar then at the lower central incisors in “ortho‐treated” group. In the upper jaw, gingival recession was present as well in the “ortho‐treated” group in the premolars with significant differences only on the right side (Figure 2).

#### 3.2.3 Difference in the number of sites with papilla fill between “ortho‐treated” and “nontreated” groups

Papilla fill had an overall fully filled sites in (52.1%) compared to (47.9%) as an overall sites with reduced papilla fill. Around 57% of sites showed a fully filled papilla in “ortho‐treated” group, while it was reduced at more sites of “nontreated” groups with (52.9%) with no significant difference detected in both of them (*p* = 0.066) (Table 3).

#### 3.2.4 Difference in the keratinized gingiva between “ortho‐treated” and “nontreated” groups

The mean scores of keratinized gingiva were around (2.89 mm) as shown in (Table 3) with a higher width of keratinized gingiva in the “nontreated” group showing a mean of (2.98 ± 0.47 mm) compared to (2.79 ± 0.39 mm) for the “ortho‐treated” group with significant difference (*p* < 0.001).

**Figure 3:**
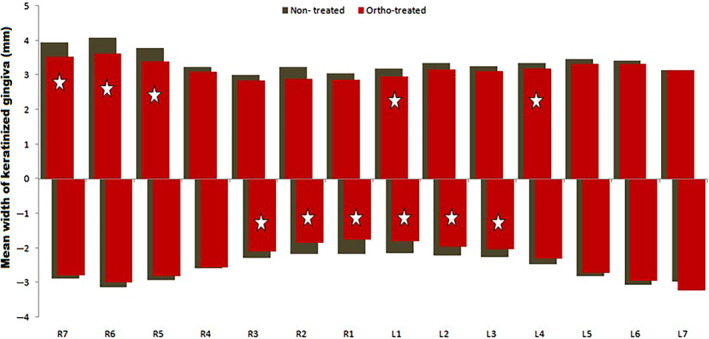
Mean of keratinized gingiva for the sample population by tooth among treatment status

It is noteworthy that a narrower keratinized gingiva was measured at the lower incisors followed by the lower canines in the “ortho‐treated” group compared to the “nontreated” group. Lower right and left central incisors showed a mean of (1.75 and 1.8 mm respectively) for the “ortho‐treated” group whereas lower right and left central incisors had a mean of (2.17, 2.15 mm respectively) for the “nontreated” group, this difference showing a narrower keratinized gingiva at the lower incisors in “ortho‐treated” compared to “nontreated” group was found to be statistically significant (Figure 3).

### 3.3 Radiographic parameters for “ortho‐treated” and the “nontreated” groups

#### 3.3.1 Changes in crestal bone level between “ortho‐treated” and “nontreated” groups

Table (4.4) shows radiographic parameters in both “ortho‐treated” and “nontreated” groups. No statistically significant difference in overall Crestal Bone Level (*p* = 0.200) was detected with a mean distance of (1.78 ± 0.20 mm) between the crest of alveolar bone and the cementoenamel junction in the ortho‐treated group and (1.75 ± 0.21 mm) in the nontreated group.

**Figure 4:**
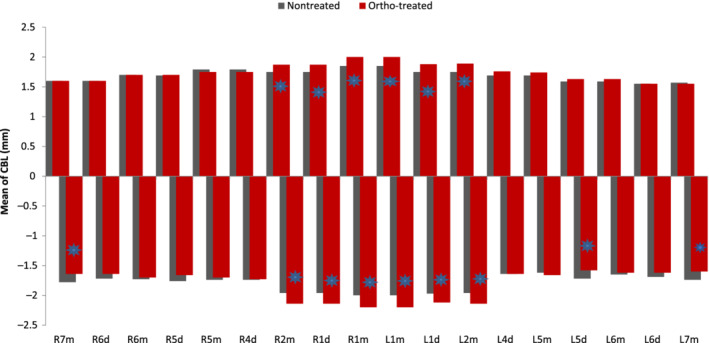
Mean of crestal bone level for the sample population by tooth among treatment status

Crestal bone level showed different patterns around the dentition. A significantly lower level of crestal bone was evident in the anterior segments in both jaws for the “ortho‐treated” group compared to “nontreated” group. It was most significant in the upper anterior segment (*p* = 0.002) compared to (*p* = 0.005) in the lower anterior segment (Table 4) and (Figure 4).

**Table 4:** Means (±*SD*) and N (%) of radiographic parameters of study sample (N = 311)

Variable^a^	Overallmean ± *SD*	Groups		*p*‐value independent *t*‐test
		Nontreated mean ± *SD*	Ortho‐treated mean ± *SD*	
Crestal bone level (overall)	1.77 ± 0.21	1.75 ± 0.21	1.78 ± 0.20	0.200
Crestal bone level for upper jaw	1.72 ± 0.22	1.69 ± 0.22	1.75 ± 0.21	0.014
Crestal bone level of right upper posteriors	1.69 ± 0.29	1.69 ± 0.31	1.69 ± 0.28	1.000
Crestal bone level of upper anterior	1.85 ± 0.38	1.78 ± 0.35	1.91 ± 0.39	0.002
Crestal bone level of left upper posterior	1.63 ± 0.29	1.61 ± 0.32	1.64 ± 0.26	0.364
Crestal bone level of lower jaw	1.81 ± 0.28	1.80 ± 0.28	1.82 ± 0.27	0.521
Crestal bone level of lower left posterior	1.66 ± 0.29	1.69 ± 0.34	1.63 ± 0.24	0.073
Crestal bone level of lower anterior	2.1 ± 0.53	1.98 ± 0.51	2.15 ± 0.54	0.005
Crestal bone level of lower right posterior	1.72 ± 0.37	1.76 ± 0.40	1.68 ± 0.33	0.055
Proximal caries	N (%)	N (%)	N (%)	*χ* ^2^–test 0.180
No	282(90.1)	135(87.1)	147(93.0)	
	31(9.9)	20(12.9)	11(7.0)	
Yes				

^a^ Means of radiographic parameters consist of (overall crestal bone level, crestal bone level for upper jaw, crestal bone level of upper right posteriors, crestal bone level of upper anterior, crestal bone level of upper left posteriors, crestal bone level of lower jaw, crestal bone level of lower left posteriors, crestal bone level of lower anterior, crestal bone level of lower right posteriors and proximal caries) between nontreated and ortho‐treated groups.

It was noteworthy that in the upper jaw a mean lower crestal bone level of (1.75 ± 0.21 mm) was evident for the “ortho‐treated” group whereas (1.69 ± 0.22 mm) for the “nontreated” group. This was a statistically significant difference (*p* = 0.014).

Both lower right and left second molars and lower left second premolar showed statistically significant differences as single tooth units comparing crestal bone level between the “ortho‐treated” and the “nontreated”, those statistically significant differences were separated from the whole overall posterior segment measurements shown in Table (4.4) as a group of teeth combined together (Figure 4).

#### 3.3.2 Prevalence of proximal caries in the “ortho‐treated” and “nontreated” groups

**Figure 5:**
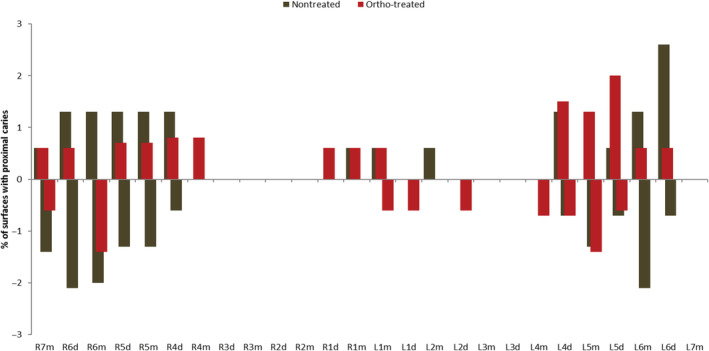
Percent of surfaces with proximal caries for the sample population by treatment status

The number of surfaces with proximal caries were more in the “nontreated” group, showing that most of “ortho‐treated” subjects surfaces were free of any signs of proximal caries, with no significant differences between the two groups (*p* = 0.180) (Table 4) (Figure 5).

### 3.4 “Extraction” versus “nonextraction” treatments in “ortho‐treated” group

Approximately one‐third (32.6%) of the “ortho‐treated” group sample had extractions for orthodontic purpose as method of treatment to improve malocclusion as shown (Table 5). As demonstrated, most of the parameters like gingival recession, smile line, tooth display and papilla fill failed to prove any significant difference between those two methods of treatment. While keratinized gingiva and number of displayed teeth showed a statistically significant difference (Table 6).

**Table 5:** Extraction by groups of study sample (N = 156)

Variable^a^	N(%)
Extraction status among ortho‐patients	
Nonextraction	105(67.3)
Extraction	51(32.6)

^a^ Extraction status among ortho‐treated group of the study sample.

**Table 6:** Means (±*SD*) and N (%) of clinical parameters of study sample by groups (N = 156)

Variable^a^	Overall mean ± *SD*	Groups		*p*‐value independent *t*‐test
		Nonextraction mean ± *SD*	Extraction mean ± *SD*	
Gingival recession	0.08 ± 0.11	0.09 ± 0.11	0.08 ± 0.10	0.402
Keratinized gingiva	2.80 ± 0.39	2.83 ± 0.39	2.70 ± 0.38	0.003
Smile line	1.15 ± 0.95	1.18 ± 0.95	1.06 ± 0.94	0.263
Tooth display length	2.1 ± 1.1	2.09 ± 1.2	2.2 ± 0.88	0.357
No. displayed teeth	5.78 ± 1.57	6.02 ± 1.49	5.28 ± 1.63	<0.001
Papilla fill	N(%)	N(%)	N(%)	*χ* ^2^–test < 0.001
Reduced	68(43.0)	45(42.1)	23(45.1)	
Fully filled	90(57.0)	62(57.9)	28(54.9)	

^a^ Mean of clinical parameters consist of (gingival recession, keratinized gingiva, smile line, length of tooth display, number of displayed teeth and papilla fill) between nonextraction and extraction sub‐groups of ortho‐treated group of the study sample.

The overall width of keratinized gingiva showed a mean of (2.80 ± 0.39 mm), with a thicker width for “nonextraction” group (2.83 ± 0.39 mm) compared to (2.70 ± 0.38 mm) for those in the “extraction” group (*p* = 0.003).

Regarding the number of displayed teeth, the overall number of displayed teeth was (5.78 ± 1.57), showing a higher number of displayed teeth in “nonextraction” groups (6.02 ± 1.49) compared to only (5.28 ± 1.63) recoded for “extraction” group. This difference between those two groups reached a statistically significant difference with *p* < 0.001 (Table 6).

### 3.5 Radiographic parameters for “extraction” versus “nonextraction” treatments in “ortho‐treated” group

Crestal bone level is an important radiographic parameter, it has an overall mean with (1.78 ± 0.20 mm), showing a very minimal difference between “nonextraction” and “extraction” group with mean of (1.79 ± 0.18 and 1.78 ± 0.24 mm respectively) (Table 7).

**Table 7:** Means (±*SD*) and N (%) of radiographical parameters by groups (N = 156)

Variable^a^	Overall mean ± *SD*	Groups		*p*‐value independent *t*‐test
		Nonextraction mean ± *SD*	Extraction mean ± *SD*	
Crestal bone level (overall)	1.78 ± 0.20	1.79 ± 0.18	1.78 ± 0.24	0.678
Crestal bone level for upper jaw	1.75 ± 0.22	1.76 ± 0.18	1.73 ± 0.29	0.274
Crestal bone level of right upper posteriors	1.69 ± 0.28	1.69 ± 0.25	1.70 ± 0.35	0.772
Crestal bone level of upper anterior	1.91 ± 0.40	1.94 ± 0.40	1.86 ± 0.39	0.075
Crestal bone level of left upper posterior	1.64 ± 0.26	1.64 ± 0.24	1.64 ± 0.31	1.000
Crestal bone level of lower jaw	1.82 ± 0.27	1.82 ± 0.25	1.83 ± 0.31	0.754
Crestal bone level of lower left posterior	1.63 ± 0.24	1.60 ± 0.17	1.70 ± 0.35	0.002
Crestal bone level of lower anterior	2.15 ± 0.54	2.2 ± 0.55	2.1 ± 0.51	0.097
Crestal bone level of lower right posterior	1.68 ± 0.33	1.67 ± 0.29	1.71 ± 0.41	0.321
Proximal caries	N(%)	N(%)	N(%)	*χ* ^2^–test 0.010
No	147(93.0)	98(91.6)	49(96.1)	
	11(7.0)	9(8.4)	2(3.9)	
Yes				

^a^ Mean of clinical parameters consist of (gingival recession, keratinized gingiva, smile line, length of tooth displayed, number of displayed teeth and papilla fill) between nonextraction and extraction subgroups of ortho‐treated group of the study sample.

A surface free of proximal caries was manifested in both “nonextraction” and “extraction” group showing (91.6% and 96.1%, respectively). The extraction group had significantly greater percentage of sites free of caries compared to the “nonextraction” group (*p* = 0.01) (Table 7).

## 4 DISCUSSION

A satisfactory functional and esthetic outcome is the main goal to be achieved in any orthodontic treatment. In this comparative cross‐sectional study functional and esthetic outcome was evaluated by comparing the effects of orthodontic treatment on the periodontium in adult patients to those who were never treated orthodontically.

Our study showed an overall mean of crestal bone level in “ortho‐treated” and in “nontreated” group comparable to other studies. This was supported by a number of studies that measured the distance between cemento‐enamel junction to alveolar crest on radiographs showing a mean of (1–2 mm) (Chapple et al., 2018; Glavind et al., 1968; Newman et al., 2011; Tarnow et al., 1992).

A statistically significant difference was observed in the crestal bone level at the anterior upper and lower teeth between “ortho‐treated” compared to the “nontreated” group radiographically. This corresponds with the possible adverse tissue reactions mentioned in earlier studies. Using bitewings radiograph to assess crestal bone level was shown to be an acceptable method by Baxter (1967).

A significant difference in the marginal bone level of middle age patients after orthodontic treatment in the anterior segment was shown in a study done by Han et al. (2019). This change in the crestal bone was confirmed in the current study in both upper and lower jaws in “ortho‐treated” group compared to “nontreated” group, showing statistically significant differences. This was explained previously by Phermsang‐ngarm and Charoemratrote (2018). When it was reported that decreased bone thickness labial to the incisors may have been due to the amount of proclination and the magnitude of forces applied to the tooth leading to bone resorption around it.

The results of our study demonstrated more gingival recession in the “ortho‐treated” however this failed to reach statistical significance (*p* = 0.120). Gingival recession was the most at lower second premolar then at lower central incisors in the “ortho‐treated” group. Yared et al. (2006) assessed periodontal status of mandibular central incisors after orthodontic proclination in adults reporting that free gingival margin thickness (<0.5 mm) had a direct relation to gingival recession and showing severe gingival recession in the mandibular central incisors after orthodontic treatment (Yared et al., 2006). They explained this relation to gingival recession with the fact that tooth advancement might induce free gingival‐margin tension, which would become narrower and thinner with movement of the teeth.

On the other hand, Kamak et al. (2015), who studied the effect of changes in lower incisor inclination on gingival recession reporting absence of any influence of orthodontic displacement of lower incisors on the development of gingival recessions in the mandibular incisor region.

Statistically significant differences in the width of keratinized gingiva were recorded for most of the upper and lower incisors between the “ortho‐treated” and the “nontreated” groups; with the first group exhibiting lower width of keratinized gingiva. This may be due to the pre‐existing width of keratinized gingiva in those teeth before having orthodontic treatment. The results reported by Coatoam et al. (1981) which showed greater incidence (6.1%) for the complete loss of keratinized gingiva on the teeth with (<2 mm) of keratinized gingiva, than on teeth with (>2) mm (0.1%) following orthodontic treatment explained and supported our findings. Changes in tooth position, frequently, can be directly related to dimensional changes in the keratinized gingiva which might account to those differences in width of keratinized gingiva (Coatoam et al., 1981).

These differences in the width of keratinized gingiva indicate that orthodontic treatment affects the width of the keratinized gingiva. Preorthodontic assessment is of great importance and if minimal width is detected changes in the width should be expected depending on the orthodontic movement. In these cases, soft tissue augmentation should be considered to prevent further reduction.

Regarding esthetic parameters, the smile line was highest in the upper lateral incisors then the centrals with significant differences between the “ortho treated” and the “nonortho” treated. However, the overall mean scores of smile line failed to show any significant difference. Those results disagreed with the conclusion by Nahm et al. (2017) that excessive gingival display could be reduced with orthodontic treatment, he reported a new bone formation on palatal side of upper anterior teeth. Teeth were moved into augmented area without fenestration or vitality loss. This resolved lip protrusion, and thus the excessive gingival display was effectively improved (Nahm et al., 2017). This can be possibly explained by the fact that a specific type of malocclusion was assessed in the Nahm et al. study.

The authors believe that having a mean gingival smile line of (1.15 mm) for “ortho‐treated” patient is accepted as the current literature suggests that an attractive smile shows between 0 and 2 mm of gingiva (40–45), and again those “ortho‐treated” patient might have had a previously higher smile line that was resolved during treatment.

Regarding the ongoing debate “extraction” versus “nonextraction” orthodontic treatment methods. Our investigation showed no significant differences between patients who underwent extraction as part of their orthodontic treatment and those without extraction, except a statistically significantly reduced width of keratinized gingiva showing less width when extraction of teeth was part of the treatment. This could have no negative sequelae if good oral hygiene and strict maintenance is established, especially that the mean width was more than 2 mm (Hangorsky & Bissada, 1980; Miyasato et al., 1977; Wennström et al., 1981). These findings are consistent with the results reported by a number of other studies looking into the possible advantages of nonextraction orthodontic treatment (Bishara et al., 1995, 1997; Bowman & Johnston Jr, 2000; Dewel, 1964; Paquette et al., 1992; Zierhut et al., 2000).

The number of teeth displayed was significantly less in the “extraction” group compared to “nonextraction” group. This might affect the smile esthetics perception according to Cheng et al. who investigated the effect of “nonextraction” and “extraction” orthodontic treatments on smile esthetics for different malocclusions. In this study, it was concluded that a smile with a higher number of displayed teeth was considered a more esthetic smile (Chen et al., 2005).

Despite the challenge orthodontic appliance might pose in performing proper oral hygiene, the prevalence of caries in the “ortho treated” group was lower than that of the “nontreated” group. This difference did not reach statistical significance This might be related to the regular visits to the orthodontist at which carious lesions can be detected and treated promptly.

The cross‐sectional design of this study and the heterogeneity of the types of orthodontic malocclusion and movements are limitations of this study. However, it still provides valuable information to periodontists and general dentist as to expected effects of different types of orthodontic treatment to be able to distinguish that from signs of active dental or periodontal disease. Future prospective longitudinal studies assessing the changes in the level of bone crest, periodontal tissues and in smile esthetics including patient‐centered outcomes would be of great value.

In the general population, orthodontic treatment appeared to be associated with minimal detrimental effects on the periodontal tissues. Minimal significant difference in the width of keratinized gingiva and number of teeth displayed was noted when extraction was part of or was avoided in orthodontic treatment. These differences might not be of great clinical significance. When assessing the crestal bone level on bitewing radiographs, patients with history of orthodontic treatment might have lower crestal bone levels in relation to the cementoenamel junction at certain sites and it is important that this minimal bone loss would not be confused with periodontal disease. Within the limits of the present study, the results indicate that, practitioners can reach an achievable outcome from orthodontic treatment in a manner that respects limits which keeps all parameters in the acceptable side. Additional longitudinal studies, randomized, and controlled clinical trials are necessary to adequately test the potential behavior of each orthodontic displacement and its effect on each esthetic and functional parameter.

## AUTHOR CONTRIBUTIONS

**R.A.** formulated the research idea and methodology and directly supervised data collection, analysis and write up. **A.T.** played a major role in patient recruitment, data collection, analysis and write up. **D.T.** provided mentorship and guidance. All authors discussed the results and contributed to the final manuscript.

## Data Availability

The data that support the findings of this study are available on request from the corresponding author. The data are not publicly available due to privacy or ethical restrictions.
